# Chromatic discrimination measures in mature observers depend on the response window

**DOI:** 10.1038/s41598-022-13129-w

**Published:** 2022-05-31

**Authors:** Julien Fars, Thiago P. Fernandes, Cord Huchzermeyer, Jan Kremers, Galina V. Paramei

**Affiliations:** 1 Department of Ophthalmology, University Hospital Erlangen, Schwabachanlage 6, 91054 Erlangen, Germany; 2 Department of Psychology, Federal University of Paraiba, Cidade Universitaria S/N, Joao Pessoa, 58051-900 Brazil; 3 Department of Psychology, Liverpool Hope University, Hope Park, Liverpool, L16 9JD UK

**Keywords:** Psychology, Cognitive neuroscience, Neural ageing, Visual system, Colour vision

## Abstract

Our past anecdotal evidence prompted that a longer response window (RW) in the Trivector test (Cambridge Colour Test) improved mature observers’ estimates of chromatic discrimination. Here, we systematically explored whether RW variation affects chromatic discrimination thresholds measured by the length of Protan, Deutan and Tritan vectors. We employed the Trivector test with three RWs: 3 s, 5 s, and 8 s. Data of 30 healthy normal trichromats were stratified as age groups: ‘young’ (20–29 years), ‘middle-aged’ (31–48 years), and ‘mature’ (57–64 years). We found that for the ‘young’ and ‘middle-aged’, the thresholds were comparable at all tested RWs. However, the RW effect was apparent for the ‘mature’ observers: their Protan and Tritan thresholds decreased at 8-s RW compared to 3-s RW; moreover, their Tritan threshold decreased at 5-s RW compared to 3-s RW. Elevated discrimination thresholds at shorter RWs imply that for accurate performance, older observers require longer stimulus exposure and are indicative of ageing effects manifested by an increase in critical processing duration. Acknowledging low numbers in our ‘middle-aged’ and ‘mature’ samples, we consider our study as pilot. Nonetheless, our findings encourage us to advocate a RW extension in the Trivector protocol for testing mature observers, to ensure veridical measures of their chromatic discrimination by disentangling these from other ageing effects—slowing down of both motor responses and visual processing.

## Introduction

Changes in colour vision are sensitive biomarkers of various health conditions; hence, its assessment is an essential part of current diagnostic tool batteries, both in clinical^1^ and academic settings (e.g., Refs.^2–4^).

The Cambridge Colour Test (CCT) is a computerised colour vision diagnostic tool developed by Mollon, Reffin and Regan^5,6^. Due to its uncomplicated way of administration and rapidness, from a testee’s viewpoint, and provision of precise chromatic discrimination measures, from the tester’s perspective, the CCT currently is used worldwide^7^. The CCT enables an accurate diagnosis of the type of colour vision impairment (Protan, Deutan, or Tritan) and its degree^6^.

In clinical practice, the CCT is employed for detection of acquired colour vision deficiencies caused by (hereditary) retinal diseases or optic nerve pathologies^8–11^, glaucoma^12^, systemic diseases such as diabetes^13–15^, exposure to neurotoxic substances^16,17^, and various neurodevelopmental conditions^18–23^.

**Figure 1:**
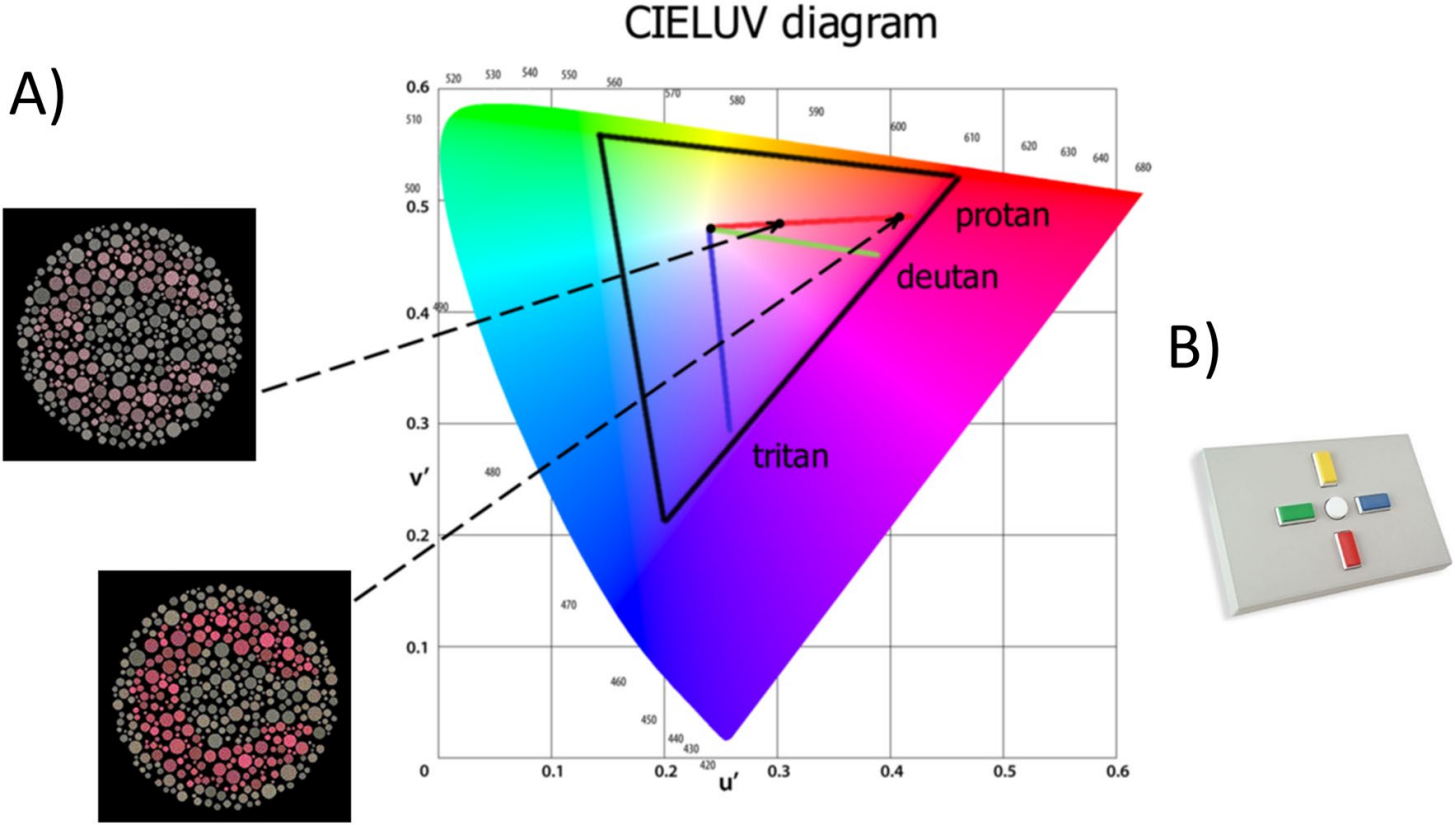
(**A**) Illustration of the chromatic targets, Landolt “C”, embedded in the luminance noise background (image source: Cambridge Colour Test Handbook (2000, p. 4) and the Protan, Deutan, and Tritan vectors in the CIE 1976 u′v′ chromaticity diagram (image source: Cambridge Research Systems Ltd., https://www.psychophysics.uk/colour-discrimination/). Permission has been obtained from Prof. John D. Mollon, who holds the copyright of the CCT. (**B**) Response box RB-540 provided by Cambridge Research Systems Ltd. and used in the experiment. Four choices are available to indicate position of the Landolt “C” opening on a given trial: up, down, right and left.

The CCT Trivector test^6^ allows estimation of chromatic discrimination thresholds along Protan, Deutan and Tritan confusion lines of the CIE u′v′ (1976) chromaticity diagram, which are mainly driven by activity of the L-, M- and S-cones respectively^6^. The target figure, a Landolt “C” ring, varies in chromaticity only and is embedded in a grey background of non-hue noise (Fig. 1A). The opening in the “C” ring can take on one of four positions, with the observer’s task to detect the direction of the “C” opening and press the corresponding response box button (Fig. 1B).

The default Trivector testing protocol, indicated in the CCT Handbook^6^, recommends a response window (RW) of 3 s for reliably testing a population of young normal trichromats. The RW implies here the duration encompassing the stimulus presentation and the testee’s response. The stimulus remains presented for the allocated time (e.g., during the default 3 s) or, if the testee responds before the end of the allocated time, disappears as soon as the response is input. If no response is given during the allocated time, the program treats the response as incorrect. Notably, the Trivector testing protocol allows extending the RW, which was recommended by Mollon and Regan^6^ for testing vulnerable populations. Indeed, longer RWs (5 s, 6 s, or 8 s) were employed for testing children^24^ and clinical populations^21–23^; in some studies, patients with significant visual impairments were allowed up to 20 s to respond^9,19^.

The RW extension conceivably is beneficial for older observers in general, to counterweigh a decrease in speed of visual processing^25,26^ and motor response^27^. While deterioration of chromatic sensitivity with age, especially after 60 years, is well documented^4,28,29^, there is some evidence that this is also accompanied by a decrease in speed of colour processing^30^.

The deterioration in chromatic sensitivity can have different causes. In the optical system of the eye, the main cause of declining chromatic sensitivity is the increasing opaqueness and yellowing of the crystalline lens that foremost affects tritan discrimination^31–34^. Ocular media senescence can account for 40% of the age loss related to the S-cones pathway, whereas the remaining loss is considered to be related to post-receptoral mechanisms^35^. At the retinal level, integrity of the S-cone mosaic and the post-receptoral S-cone pathway are implicated^36–39^. Also, losses in chromatic discrimination based on L- and M-cone activity were attested^36^. In addition, a decline in neural efficiency of colour processing at the cortical level was demonstrated^40,41^.

In our earlier studies using the CCT Trivector test^4,42^, we observed that mature normal trichromats (those in their 60 s and older) were challenged by the 3-s RW, whereby frequently their motor reaction was too slow and/or, during the short allocated time, they hesitated about the response choice. To overcome the problem of spurious incorrect discrimination responses (recorded as such by the CCT when no response was input), we allocated a longer response time of 8 sec^4,42^.

Noteworthy, Shinomori et al.^34^, too, extended the RW to 5 s remarking that some older participants struggled to respond within 3 s. Further, Dees and Baraas^3^ suggested that the 3-s RW protocol used in their study might have resulted in normal trichromats’ Trivector thresholds that were higher than the normative data reported by Paramei and Oakley^4^ obtained with an 8-s RW protocol.

The aim of the present study was to systematically probe the response window—3 s, 5 s, or 8 s—to explore whether the temporal variation in the Trivector protocol affects chromatic discrimination measures in healthy participants with normal colour vision. In addition to the RW analysis for the whole participant sample, we studied possible RW effects for age groups within it.

An auxiliary aim was assessing Trivector repeatability at the three RWs by testing each participant twice; We gauged of coefficients of repeatability Trivector estimates by those reported earlier^43,44^.

## Results

**Figure 2:**
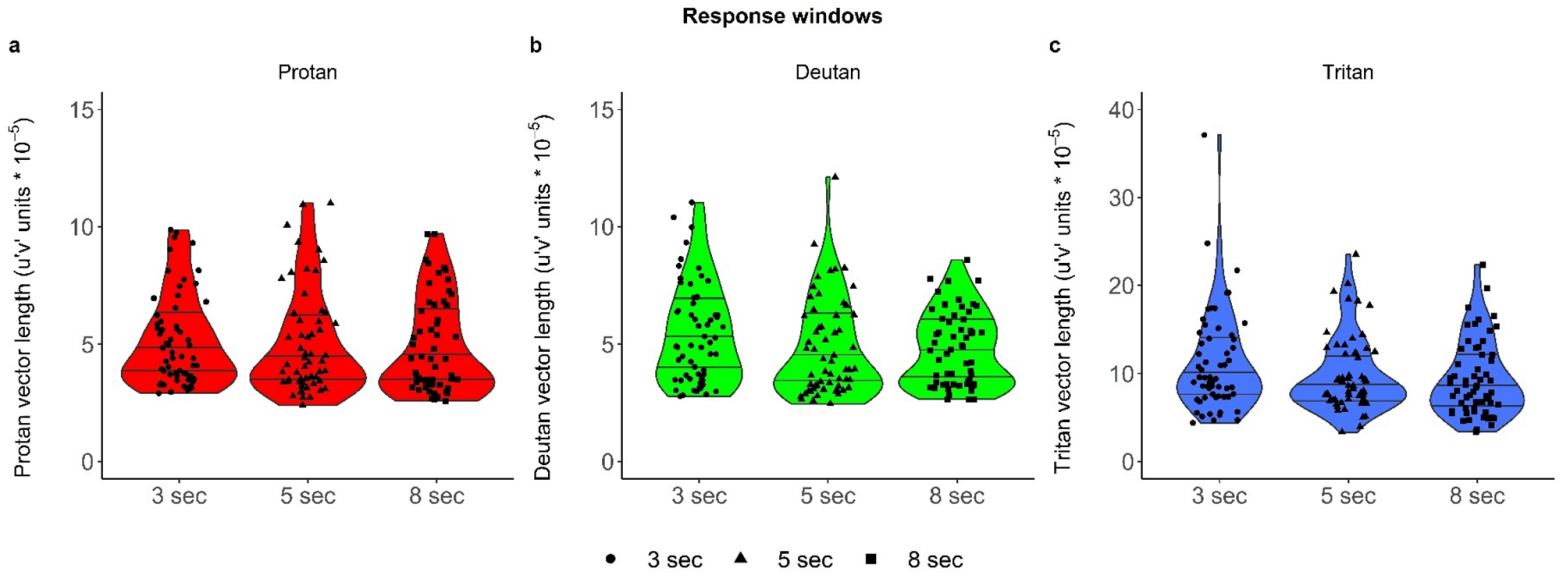
Violin plots representing discrimination thresholds of individual observers at the 3-s, 5-s and 8-s response windows for each of the three Trivector measures: (**a**) Protan, (**b**) Deutan and (**c**) Tritan. Observe that the y-axis scale of Tritan thresholds (**c**) differs from those of Protan (**a**) and Deutan (**b**) thresholds. Horizontal lines denote 25%, 50% and 75% quartiles. The shape of the violin plots is determined using a Gaussian kernel density.

Descriptive statistics of the Trivector thresholds for each of the three RWs, for the whole participant sample are shown in Table 1 and illustrated in Fig. 2. In Fig. 2 one participant exhibited a large Tritan value at 3-s RW. This datapoint collected during the test session comes from the numerous absences of response, considered as incorrect by the CCT program. During the retest session, the participant displayed less absence of responses but some of them were traded for incorrect responses. Consequently, the second highest value of the Tritan 3-s RW dataset comes from the same participant.

**Table 1:** Descriptive statistics of the Trivector measures (10^–5^ u′v′ units) for 3-s, 5-s and 8-s response windows, for test and retest, for the sample of normal trichromats (N = 30): Mean, standard deviation (SD), median (Mdn), semi-interquartile range (sIQR), and skewness. Outcomes of the Shapiro–Wilk test (p-value) indicate that the majority of data were not normally distributed; in three cases (indicated in bold) p-value was marginally significant.

Response window	3 s		5 s		8 s	
Test order	Test	Retest	Test	Retest	Test	Retest
**Protan**						
Mean	5.239	5.082	5.110	4.910	5.145	4.564
SD	2.050	1.845	2.302	2.112	2.202	1.786
Mdn	4.659	4.523	4.324	3.836	4.380	3.600
sIQR	1.508	1.085	1.489	1.207	1.700	1.316
Skewness	0.946	1.031	1.191	1.233	0.735	0.878
p-value	0.003	0.005	0.001	< 0.001	0.003	0.001
**Deutan**						
Mean	5.620	5.177	4.888	4.672	5.044	4.477
SD	2.355	1.824	2.185	1.903	1.717	1.378
Med	4.874	5.409	4.183	3.925	5.135	4.036
sIQR	1.907	1.361	1.209	1.581	1.530	1.068
Skewness	0.772	0.579	1.466	0.841	0.224	0.740
p-value	0.012	0.027	0.001	0.002	**0.052**	0.026
**Tritan**						
Mean	10.980	11.211	9.846	9.755	9.439	9.277
SD	6.537	4.724	4.238	4.271	4.411	4.139
Med	8.822	10.860	8.305	8.675	7.921	8.551
sIQR	3.411	2.653	1.828	2.733	3.026	2.491
Skewness	2.419	1.047	1.320	1.339	0.690	1.359
p-value	< 0.001	**0.060**	< 0.001	0.005	**0.069**	0.003

**Figure 3:**
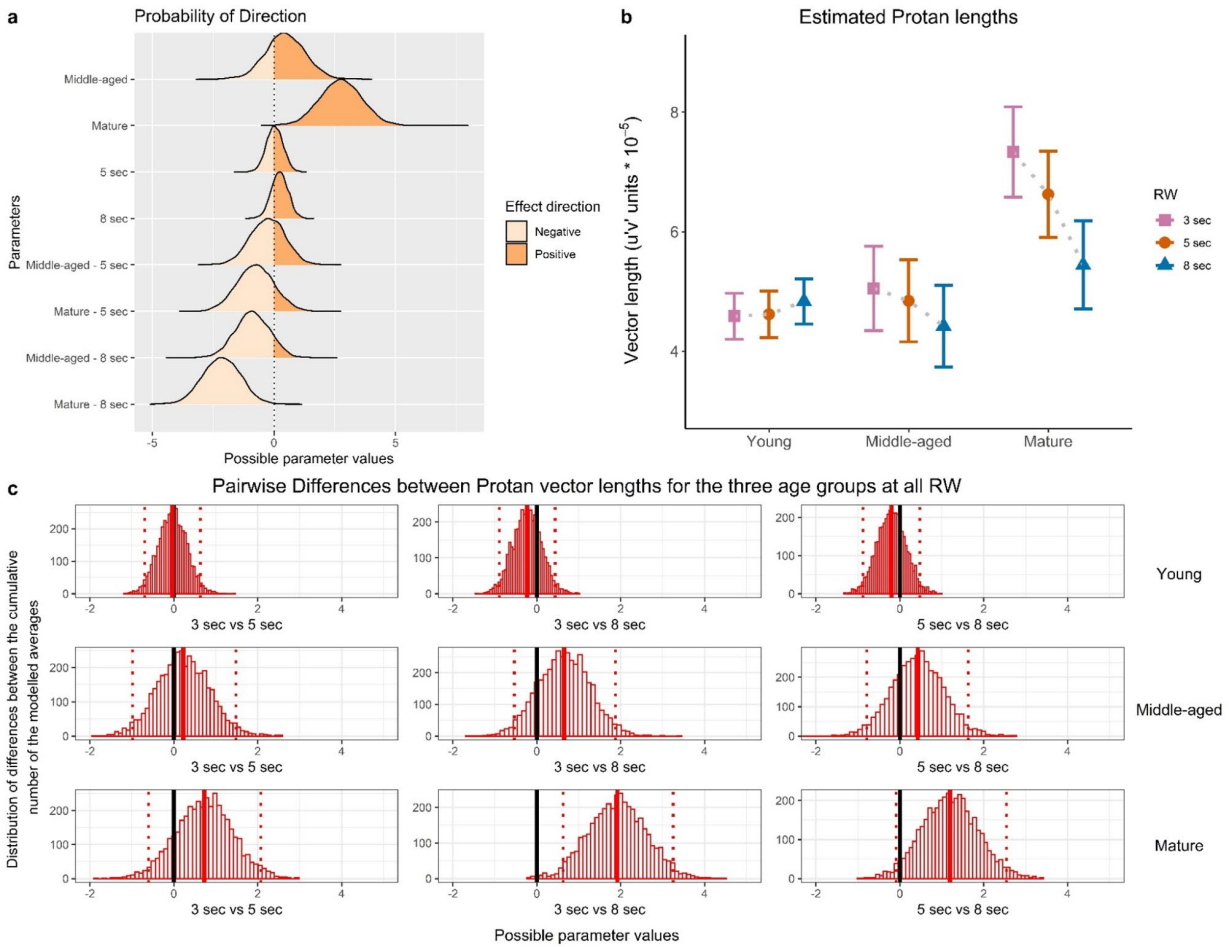
Protan measures. Outcomes of the Bayesian model of analysis showing the effect of the response window (RW) for ‘young’, ‘middle-aged’ and ‘mature’ observers. (**a**) The Probability of direction (Pd), positive or negative, of each of the model parameters. Parameters are compared to their references: ‘middle-aged’ and ‘mature’ groups are compared to the ‘young’ group, 5-s and 8-s RWs are compared to 3-s RW etc. Positive direction indicates higher values and negative direction lower values compared to the reference group. (**b**) Estimated vector length (mean and 95% CI) based on the posterior fit of the Bayesian model. (**c**) Pairwise differences of the estimated lengths for each of the age groups between the three RWs (3 s, 5 s, 8 s). Distributions of the modelled average differences were generated by substracting vector length at a longer RW from that at a shorter RW, whereby a positive distribution indicates greater vector lengths at the shorter RW. The distribution mean is indicated by thick red line, and the uncertainty interval (2.5–97.5%) by dashed lines; where the uncertainty interval does not contain 0 (thick black line), the difference between the two measure distributions indicates that the alternative hypothesis is to be accepted.

**Figure 4:**
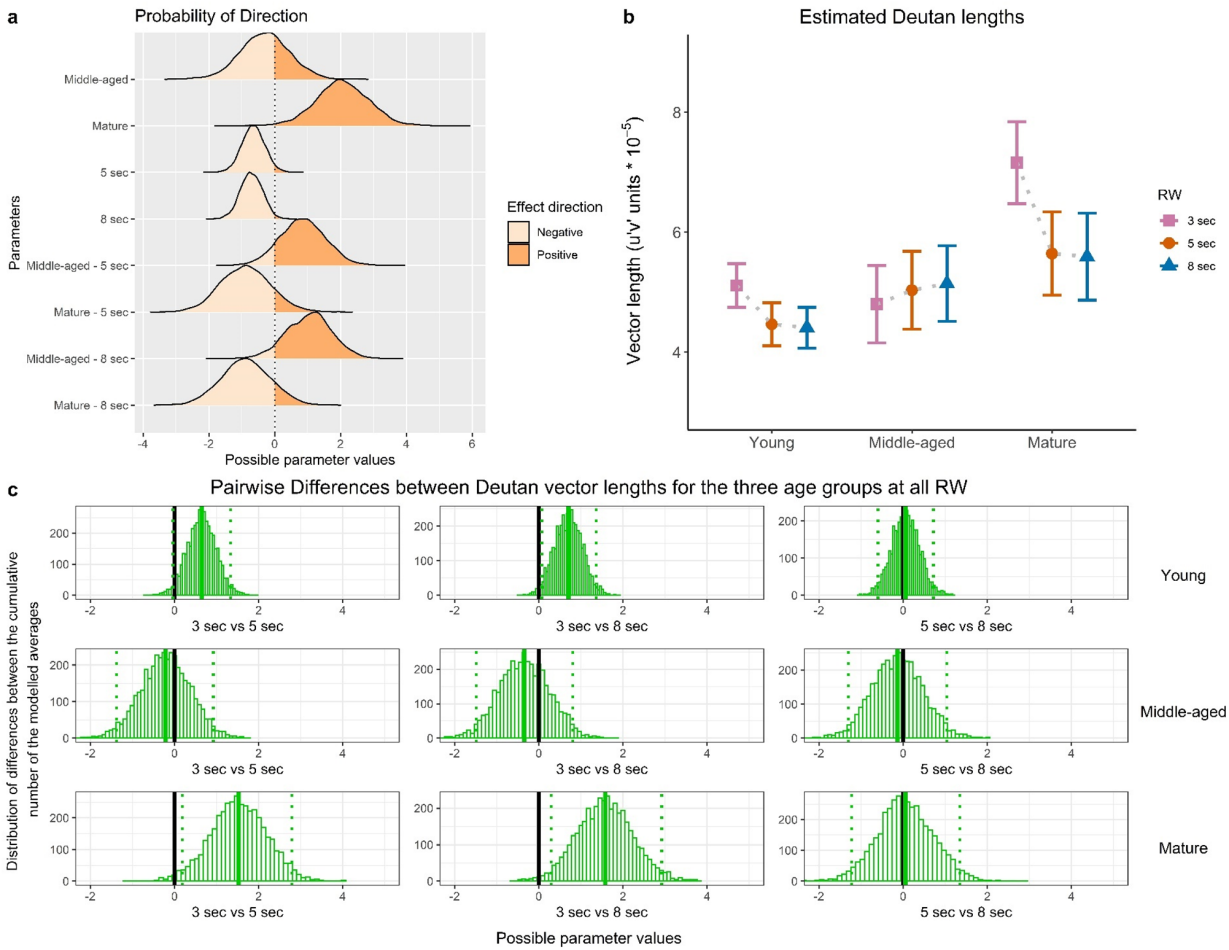
Deutan measures. The figure is organised as this figure.

**Figure 5:**
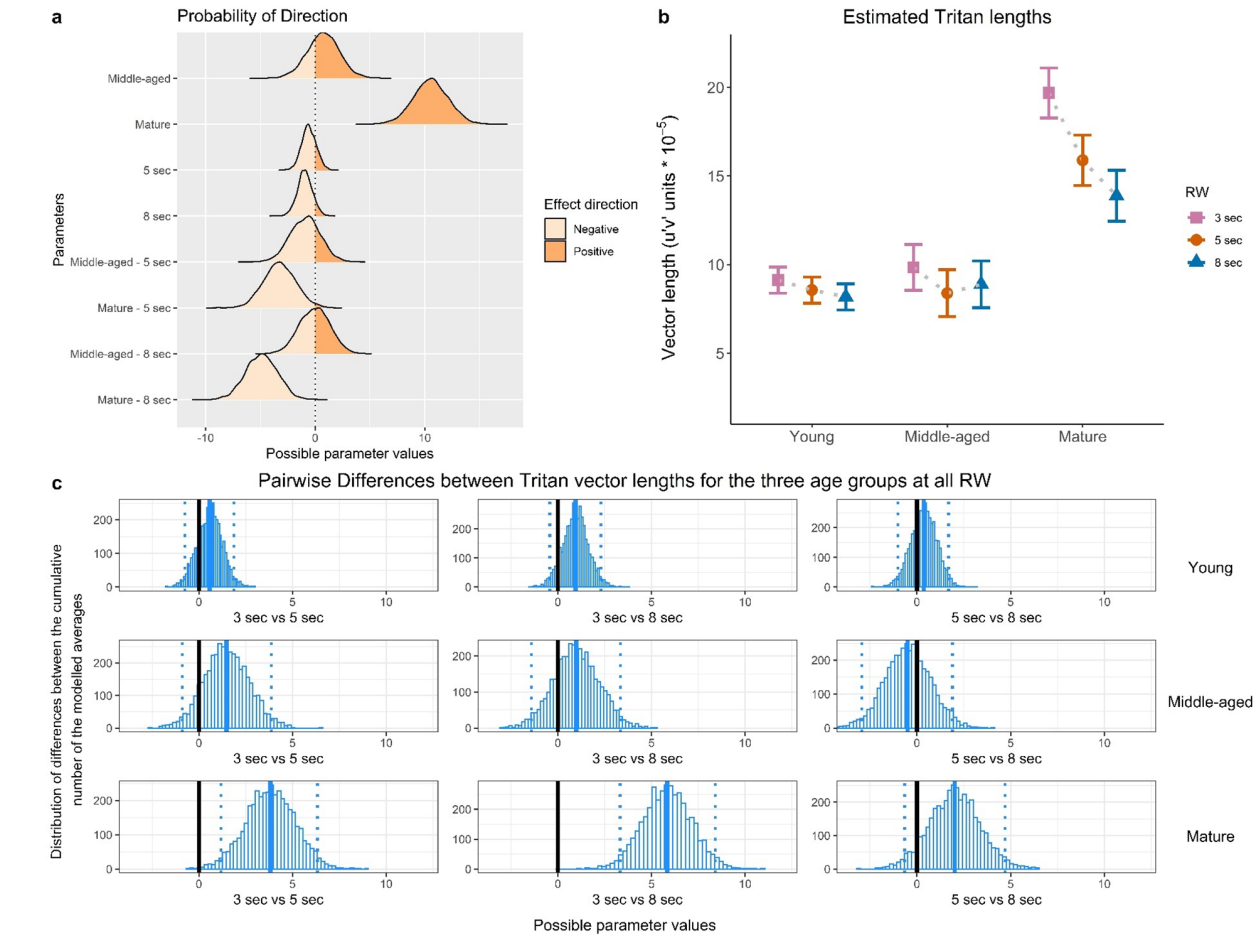
Tritan measures. The figure is organised as Fig. 4.

Table 2 shows descriptive statistics for each of the age groups. Compared to the two younger groups, the ‘mature’ group revealed higher thresholds for all three chromatic systems^4,42^, regardless of the RW duration. (Note that mean values are accompanied by CIs, forestalling report of Bayesian mixed-model analysis, whose outcomes are presented in Figs. 3, 4 and 5 and in Supplementary Table S4 of Supplementary Materials). For 3-s RW, our ‘young’ participants’ Trivector measures (means Table 2, test session) are comparable with the measures for 20–29 y.o. reported by Ventura et al. (2003; Table 34.1, combined data)^45^: for Protan, *M* = 4.4 vs. *M* = 4.3; for Deutan, M = 4.8 vs. *M* = 4.7; for Tritan, *M* = 8.3 vs. *M* = 6.7. [Our Tritan mean is slightly higher but is below the upper tolerance limit (*UL* = 11.3), in Ventura et al.’s study^45^].

**Table 2:** Mean and CIs of Trivector thresholds (10^–5^ u′v′ units) for 3-s, 5-s and 8-s response windows, for test and retest, for participants of each of the three age groups: ‘young’ (N = 19), ‘middle-aged’ (N = 6) and ‘mature’ (N = 5). Log-transformation of raw data was used for calculation means and CIs, with the following back-transformation to u′v′ units presented here.

Response window		3 s		5 s		8 s	
Test order		Test	Retest	Test	Retest	Test	Retest
**Protan**							
Age group	‘Young’	4.415 3.209–6.074	4.375 3.274–5.847	4.531 3.022–6.794	4.187 3.139–5.584	4.536 2.986–6.889	4.415 3.013–6.469
	‘Middle-aged’	4.889 3.380–7.071	4.707 3.284–6.746	4.411 2.565–7.584	4.166 2.643–6.567	4.792 2.959–7.760	3.449 2.659–4.473
	‘Mature’	7.271 5.312–9.954	6.966 5.355–9.061	5.372 4.442–7.396	6.945 4.655–10.360	5.458 3.831–7.776	4.831 3.360–6.945
**Deutan**							
Age group	‘Young’	4.855 3.360–7.015	4.778 3.391–6.733	4.314 3.016–6.172	4.100 2.852–5.894	4.336 2.962–6.347	4.039 3.065–5.323
	‘Middle-aged’	4.527 2.992–6.848	4.464 3.034–6.567	4.424 2.552–7.668	4.442 2.795–7.057	5.523 4.063–7.508	4.323 3.096–6.038
	‘Mature’	7.822 5.612–10.903	5.859 4.023–8.534	5.398 3.611–8.069	5.228 3.550–7.698	5.646 4.707–6.773	5.323 4.023–7.043
**Tritan**							
Age group	‘Young’	8.322 5.732–12.085	8.917 6.184–12.858	8.158 5.686–11.705	8.256 6.404–10.644	7.330 4.678–11.485	7.807 5.344–11.404
	‘Middle-aged’	8.697 6.373–11.870	10.381 7.660–14.069	8.802 7.456–10.392	7.142 4.242–12.025	8.619 5.720–12.988	7.846 4.870–12.642
	‘Mature’	19.846 13.681–28.789	17.868 13.791–23.150	14.512 9.915–21.242	16.120 12.794–20.512	14.469 10.902–19.202	12.730 10.570–15.333

For 5-s RW, the ‘young’ participants’ mean Trivector estimates are comparable with those reported by Shinomori et al. (2016; Table 1)^34^ for their 16–29 y.o. observers for the same RW: for Protan, *M* = 4.5 vs. *M* = 4.7; for Deutan, *M* = 4.3 vs. *M* = 4.6; for Tritan, *M* = 8.2 vs. *M* = 7.2. Our ‘mature’ group’s means are comparable, too, with those for Shinomori et al.’s group of participants in their 60 s: for Protan, *M* = 5.4 vs. *M* = 6.6; for Deutan, *M* = 5.4 vs. *M* = 6.8; for Tritan, *M* = 14.5 vs. *M* = 12.0.

### Exploring effects of the response window for the whole sample

Outcomes of the three-way (RW, Vector, Test Order) repeated-measures Bayesian ANOVA are presented in the Supplementary Materials (Supplementary Table S1). These are characterised, in the first instance, by the Bayes Factor (BF) and the Probability of direction (Pd), i.e. certainty of the effect direction. The BF estimate provides strong evidence for the effect of Vector: BF > 1000. Post hoc analysis (Supplementary Table S2) indicated that Tritan thresholds were higher than those along Protan (*Mdn* = 0.677, 95% CI = [0.61; 0.74], Pd = 100%, BF > 1000) and Deutan vector (*Mdn* = 0.678, 95% CI = [0.61; 0.74], Pd = 100%, BF > 1000). No differences were found between test and retest measures (BF = 0.140), or between the RWs (BF = 0.548). No interaction effects were present between RW and Vector (BF = 0.013), RW and the Test Order (BF = 0.048), Vector and the Test Order (BF = 0.057), or between the RW, Vector and the Test Order (BF = 0.025).

### The relationship between Trivector measures and the participants’ age

Outcomes of Bayesian analysis of correlation between Trivector measures of discrimination thresholds and participants’ age are presented in Table 3 and illustrated by Supplementary Fig. S2. In Table 3, highlighted by bold are correlation coefficients (ρ) with Pd of 98–100%, and are accompanied by sufficiently high BF-values. (BF < 1 indicates larger evidence for the null hypothesis; conversely, BF > 3 is considered to be sufficient evidence for the alternative hypothesis). It is apparent that for 3-s RW, almost all Trivector thresholds were positively correlated with age. In comparison, for the two longer RWs, 5 s and 8 s, essentially only Tritan thresholds correlated with age. Noteworthy, at 5-s RW also Protan retest thresholds correlated with age.

**Table 3:** The relationship between the vector length and age, for each response window condition, for test and retest. Presented are Bayesian correlation coefficients (ρ), Probability of direction (Pd), i.e. certainty of the effect direction, accompanied by Highest Density Intervals (95% HDI), and the Bayes Factor (BF). Correlations with Pd = 98–100%, accompanied by sufficiently high BF, are given in bold.

Vector		Protan		Deutan		Tritan	
Response window	Statistic	Test	Retest	Test	Retest	Test	Retest
3 s	Correlation coefficient	**ρ = 0.41**	**ρ = 0.37**	ρ = 0.32	ρ = 0.15	**ρ = 0.52**	**ρ = 0.50**
	Pd	**100%**	**99%**	97%	82%	**99%**	**100%**
	95% HDI	**[0.11; 0.68]**	**[0.07; 0.64]**	[0.00; 0.59]	[− 0.17; 0.47]	**[0.26; 0.77]**	**[0.26; 0.73]**
	BF	**8.110**	**5.594**	2.359	0.623	**78.651**	**50.686**
5 s	Correlation coefficient	ρ** = **0.15	**ρ = 0.42**	ρ** = **0.17	ρ** = **0.18	**ρ = 0.40**	**ρ = 0.42**
	Pd	81%	**100%**	85%	86%	**100%**	**100%**
	95% HDI	[− 0.18; 0.45]	**[0.11; 0.66]**	[− 0.13; 0.49]	[− 0.14; 0.48]	**[0.11; 0.67]**	**[0.13; 0.66]**
	BF	0.585	**10.191**	0.694	0.699	**7.884**	**12.478**
8 s	Correlation coefficient	ρ** = **0.10	ρ** = **0.01	ρ** = **0.19	ρ = 0.25	**ρ = 0.42**	**ρ = 0.43**
	Pd	73%	52%	86%	94%	**100%**	**100%**
	95% HDI	[− 0.22; 0.41]	[− 0.32; 0.32]	[− 0.13; 0.49]	[− 0.06; 0.53]	**[0.12; 0.68]**	**[0.15; 0.67]**
	BF	0.484	0.401	0.773	1.165	**9.602**	**11.475**

### Trivector measures of the ‘mature’ group are sensitive to variation of the response window

We present Bayesian model estimates of the RW effects for individual age groups and summary statistics of parameters: medians, Highest Density Intervals (95% HDI), and Probability of direction (Pd, certainty of the effect direction). Raw model output data are available in the Supplementary Materials (Supplementary Table S4). Outcomes of the model for Protan measures are illustrated by Fig. 3, for Deutan measures by Fig. 4 and for Tritan measures by Fig. 5.

We found that ‘mature’ group revealed higher threshold estimates compared to the younger groups, regardless of the RW: Protan (estimate = 2.75, 95% HDI = [1.03; 4.30], Pd = 99.90%), Deutan (estimate = 2.07, 95% HDI = [0.57; 3.56], Pd = 99.30%) and Tritan (estimate = 10.56, 95% HDI = [7.27; 13.62], Pd = 100%). Deutan thresholds for the ‘mature’ did not differ between the RWs: at 8 s (estimate = − 0.88, 95% HDI [− 2.30; 0.57], Pd: 88.55%) and 5 s (estimate = − 0.89, 95% HDI = [− 2.30; 0.61], Pd: 88.40%).

However, a decrease of Trivector thresholds was found for ‘mature’ observers at longer RWs, specifically, for Protan at 8 s (estimate = − 2.17, 95% HDI = [− 3.59; − 0.56], Pd: 99.83%); Tritan at 5 s (estimate = − 3.22, 95% HDI = [− 6.42; − 0.42], Pd: 98.08%); and Tritan at 8 s (estimate = − 4.90, 95% HDI = [− 7.85; − 2.02], Pd: 99.90%).

For ‘mature’ observers, for each of the three vectors we performed pairwise comparisons between the Trivector thresholds at the three RWs; the outcomes are shown in Figs. 3c, 4c and 5c and highlight model parameters. In particular, for Protan estimates, there is a 99.8% chance of increase when 8-s RW condition is compared to 3-s RW: *M* = 1.94, *SD* = 0.678, CI = [0.605; 3.27]. For Tritan estimates, a 99.8% chance of increase is found between 5-s and 3-s RW: *M* = 3.80, *SD* = 1.35, CI = [1.18; 6.46], and a 99.9% chance of increase between 8-s and 3-s RW: *M* = 5.83, *SD* = 1.36, CI = [3.24; 8.51]. In addition, the model revealed a small decrease of Deutan estimates at 8-s RW compared to 3-s: (estimate = − 0.71, 95% HDI = [− 1.38; − 0.01], Pd: 97.58%).

In summary, these results indicate that (i) variation of the RW does not result in any differences between Trivector thresholds of the ‘young’ and ‘middle-aged’ groups. (ii) In contrast, for the ‘mature’ group we observe the RW effect, namely: 8-s RW testing protocol resulted in lower estimates of Protan and Tritan measures, compared to 3-s RW; the Tritan measure decrease persisted at 8-s compared to 5-s RW. (iii) The ‘mature’ group’s Tritan thresholds were higher than those of the two younger groups regardless of the RW.

## Discussion

The results of the present study are in agreement with previous reports that normal trichromats reveal higher Tritan than Protan and Deutan thresholds^4,6,34,43,45^ for all RWs. Higher Tritan discrimination thresholds conceivably are related to the finding that S-cone contrast sensitivity is approximately 8 times weaker than L–M contrast sensitivity^46^. Furthermore, as argued by Nunez et al.^47^, the fundamental spatial frequency that is optimal for S-cone responses is lower than for L–M responses; in other words, to obtain S-cone responses comparable to L–M responses, a pattern should be spatially coarser. In relation to spatial characteristics of the CCT pattern, made up of small circles (i.e. elements of relatively high spatial frequency), Nunez et al.’s^47^ finding may imply that the target “C”, and, hence, the direction of the opening in it, is likely to be detected by the S-cone system sub-optimally compared to L- and M-cone systems, especially at low chromatic contrasts. The main aim of the present study was to explore the possible effect of variation of the response window on chromatic discrimination thresholds in normal trichromats along the Protan, Deutan and Tritan confusion lines. In addition, we estimated possible age and age-group effects, as well as Trivector repeatability. We discuss the findings and, also, address potential mechanisms that underlie the found RW effect for mature observers.

First, we consider the ageing effect on the Trivector thresholds. In agreement with previous studies^4,28,29^, the correlation analyses (Supplementary Fig. S2 and Table 3) showed an increase of Tritan thresholds with age for all tested RWs that probably reveals the tritan senescence effect caused by changes in the ocular media^31–34^, as well as receptoral, post-receptoral and cortical changes in the S-cone pathway^35–41,48^.

The main and novel finding of the present study is that the Trivector thresholds in mature normal trichromats, those in their 50 s and 60 s (see Fig. 3) were progressively lower at 5- and 8-s RWs than at 3-s RW. In comparison, variation of the RW did not result in any differences in Trivector thresholds of the ‘young’ (in their 20 s) or the ‘middle-aged’ (in their 30 s and 40 s).

The RW effect for Tritan thresholds in ‘mature’ observers’ is in agreement with the findings of Knoblauch et al.^28^, who employed the computerised Colour Assessment and Diagnosis (CAD) test. We compared Tritan estimates in the present study with the normative data. Notably, the present Tritan mean estimates (see Table 2) obtained at 8-s RW were within upper tolerance limits (*UL*) reported previously by Paramei and Oakley^4^: for 50+, *UL* = 15.9 and for 60+, *UL* = 13.9; but exceeded these at both 3-s and 5-s RW. However, Tritan estimates exceeded the limits reported, for 4 (out of 5) ‘mature’ observers at 3-s RW and for 3 at 5-s RW^4^. The conclusion, on the basis of Tritan thresholds at short RWs, might lead to incorrect conclusions about the involvement of pathological processes.

Several mechanisms and loci in the visual system can possibly underlie the elevation of the Trivector estimates at the shorter RWs in ‘mature’ observers. We consider these in the framework of Whiting’s^49^ model of information processing which includes the following stages: receptor systems, perceptual mechanisms, translatory mechanisms, and an effector component. We address possible causes of the RW effect at these individual stages.

The effector component is the most apparent locus of slowing of the response with age^27^. Noteworthy, along with a slower motor response, also reported was a longer preparatory interval in older observers^50^, which can be related to Whiting’s “translatory mechanisms” stage^49^. The impact of natural ageing was demonstrated specifically on perceptual decision making, in particular, in tasks demanding an increase in attention: older adults reveal a higher concern about accuracy, i.e. more conservative decision criteria, and display longer non-decisional processing times than younger adults^51^. This speed-accuracy trade-off^52^ could be due to the increased value that older observers attribute to accuracy instead of speed. The effect is more pronounced when participants can adjust their decision criteria^53^, which probably was the case in view of the present experiment’s instruction that emphasised accuracy over the speed.

Further, the sensory component and perceptual mechanisms, two other stages of information processing, are likely to show age-related slowing of visual processing^25^. Many older adults require longer response time than younger adults to detect, discriminate, recognise or identify visual targets^26,54^. As demonstrated by Ebaid and Crewther^55^ implicated in visually-driven cognitive tasks is oculomotor function, whose temporal parameters vary: in older observers, saccade durations are significantly longer than in younger observers, which affects efficient attentional processing of a stimulus and, thus, requires longer inspection time for accurate performance. The authors also conjecture that a pattern of visual fixations and their durations in older adults suggest utilising slightly different temporal strategies that, too, contribute to slower processing of complex visual stimuli.

General age-related slowing of visual processing is in agreement with findings of elevated thresholds specifically for visually complex stimuli, whose shape is defined by contrast—as is the case of the CCT stimuli in the present study—requiring more complex and simultaneously engaged networks^30,56–58^.

The temporal changes in our study were in the order of seconds whereas early chromatic changes are in the order of milliseconds. We therefore discard these mechanisms as an explanation for our results.

Finally, we shortly address the auxiliary aim of the present study—assessing test–rest stability of the Trivector thresholds at the varying RWs. For the whole participant sample, we found good repeatability of the Trivector thresholds regardless of the time allowed for the response, with no effect of the Test Order in ANOVA (although retest measures seem to have a somewhat lower dispersion; see Table 2 and Supplementary Fig. S1). The Bland–Altman analysis^59^ showed that mean test–retest differences only slightly deviated from zero for all three RWs and Trivector thresholds (see Supplementary Table S6 and Supplementary Fig. S2 of Supplementary Materials), thus, indicating the absence of a systematic learning effect, in accord with the previous findings demonstrating that CCT measures are not affected by learning^44,60^. Moreover, at least 95% of test–retest differences lie between the upper and lower LoAs for almost all datasets, in accord with the requirements of the British Standards Institution^61^.

We are aware of the main limitation of the study—a relatively small participant sample and small numbers of those comprising the ‘middle-aged’ and ‘mature’ groups; hence, the present study should be viewed as a pilot. Moreover, although it is considered that Bayesian framework can have better results than Frequentist statistics when dealing with small sample sizes, the type of prior can be crucial. Choosing priors that do not represent the sample could lead to biases in the results^62^, which is why we used weakly informative priors^63^ and investigated our data using simulations (see Supplementary Fig. S4). A future study would gain from collecting data from a more representative, age group-balanced sample of participants and, if possible, from including those in their 70 s. In addition, we cannot exclude that an extension of the RW beyond 8 s for testing mature normal trichromats would further improve their chromatic discrimination measures, particularly for Tritan vectors. Probing slightly longer RWs is worth undertaking and may enable establishing the optimal response window, beyond which chromatic sensitivity would not improve for older observers.

The present findings implicate that when the CCT (and probably other computerised tests of colour vision diagnostics) is employed, attaining veridical measures of chromatic discrimination for mature normal trichromats sensibly long RWs are required (e.g. 8 s as tested here). This would enable disentangling measurement of chromatic discrimination from the confounding ageing effects. Moreover, sufficiently long response window in the CCT protocol may be crucial in clinical practice for assessment of colour vision impairments in older patients.

## Methods

### Participants

Participants (N = 30, 18 females) were volunteers recruited from staff and students at the Universität of Erlangen-Nürnberg. The participants’ age ranged between 20 and 64 years (32.8 ± 14.1); they were grouped according to age: ‘young’ (N = 19; 20–29, M = 24.2 ± 2.7 years); ‘middle-aged’ (N = 6; 31–48, M = 36.8 ± 6.8 years); and ‘mature’ (N = 5; 57–64, M = 60.6 ± 2.6 years).

All participants had normal or best corrected visual acuity and self-reported no ocular or systemic diseases that could have affected colour vision^1^. Almost all participants (N = 26), including all ‘middle-aged’ and ‘mature’ observers, were screened for congenital red-green deficiency using the Rayleigh equation of the Heidelberg multi-color anomaloscope (Fa. Oculus). Their anomalous quotients varied between 0.72 and 1.1, i.e. within the normal range between 0.69 and 1.39^64^. The outstanding four participants self-reported normal colour vision.

Furthermore, post-data collection to rule out potential colour vision deficiency, we compared individual participants’ Trivector measures, obtained at 8-s RW, to normal trichromats’ normative data for the corresponding life decades obtained, too, at 8-s RW (Paramei and Oakley, 2014, Table 2, p. A377)^4^. We found that Trivector measures of all our participants were within previously reported tolerance limits, which verified our classification of the participants as normal trichromats.

Each observer participated with informed consent. All procedures followed protocols approved by the Faculty of Medicine of the University of Erlangen-Nuremberg. The study was conducted in accordance with the Code of Ethics of the World Medical Association of the Declaration of Helsinki.

### Apparatus

The Cambridge Colour Test v2.3.1 was employed, part of the Metropsis toolbox for psychophysical assessment of visual functions^6^ [Cambridge Research Systems Ltd. (CRS)]. Implementation and calibration procedures were performed with software and hardware provided by the CRS (OptiCAL; Bits# Stimulus Processor). Stimuli were presented on a gamma-corrected 32″ LCD Display++ monitor with 1920 × 1080-pixel resolution and frame rate 120 Hz. The monitor, with an in-built sensor system, enabled self-calibration of the display in real time that preserved maximum accuracy of the test images across testing sessions.

### Trivector test: stimuli and chromatic discrimination measures

The Trivector test estimates discrimination thresholds mainly driven by the L-, M-, and S-cones^6^. The CCT stimulus (Fig. 1A) is a pattern composed of distributed small circles randomly varying in luminance (between 6 and 16 cd/m^2^ with 2 cd/m^2^ increments) and size (between 2.8 and 5.7 arcmin in diameter). The target Landolt “C” is defined solely by a chromatic contrast; the “C” is superimposed on the noisy achromatic background, specified by u′ = 0.1977, v′ = 0.4689 (CIE 1976 chromaticity diagram). Chromatic contrast of the target varies along three confusion lines: Protan (copunctal point u′ = 0.678, v′ = 0.501), Deutan (copunctal point u′ = − 1.217, v′ = 0.782), and Tritan (copunctal point u′ = 0.257, v′ = 0.0) (Fig. 1A). The “C” opening subtends 1° of visual angle at 1.5-m viewing distance and has one of four orientations: top, bottom, left, right. To discriminate the “C”-shaped target from the background and identify the direction of the gap, the observer can only use chromatic cues and cannot rely on luminance differences or spatial cues.

In randomised Trivector presentations, the “C” chromaticity varies to reduce the contrast with the background. For each of the three confusion lines, the variation of chromatic contrast is exerted in a random order using an adaptive staircase procedure. The test stops after six staircase reversals for each vector; the chromatic discrimination threshold (in u′v′ units) is computed as the average of the chromaticity corresponding to the six reversals (For further details of the Trivector algorithm see Refs.^6,44,65^).

### Procedure

Participants were dark adapted for at least 10 min^66^ and were tested monocularly with their best eye. They were instructed to identify the orientation of the “C” gap, in one of the four positions, and press the corresponding button of the response box (Cedrus RB-540 Response box, CRS) (Fig. 1B). The participants were also instructed to press any button in case they were unable to see the “C” and/or its opening. Accuracy over speed was emphasised in the instruction. The response box, placed on participant’s lap, was held by both hands, and thumbs were used for button pressing. The stimulus was presented during the RW and remained on-screen until the button was pressed. Cue sounds indicated the start and the response input or, when no response was provided, the end of the RW. When the RW elapsed, a constant interstimulus interval (0.5 s) followed, ensued by the next trial. Absence of the response input during the RW was recorded by the CCT system as an incorrect response.

Each of the observers participated in three sessions with the varying time allowed to respond—either 3 s, or 5 s, or 8 s. The sessions varying in the RW followed directly one after another, with short breaks between these. The order of the 3-, 5- and 8-s sessions was randomised and counterbalanced among participants. The total testing time of a participant took about 20–25 min. The retest sessions were identical to the test ones and spaced of around two weeks (an exception were five participants, who completed test and retest on the same day). Testing results were recorded in the Metropsis app in tabular and graphic format, with the Trivector measures in 10^–5^ u′v′ units.

### Analysis

For each vector, Protan, Deutan and Tritan, descriptive analysis was carried out for the whole participant sample and for each of the three age groups. Since most datasets (15 out of 18) were not normally distributed, for inferential statistics data were log-transformed for application of Bayesian ANOVA and correlation analyses.

For the whole participant sample, we compared the Trivector measures using *three-way repeated-measures Bayesian ANOVA*, with three factors: RW (3), Vector (3), and Test Order (test and retest). Post hoc testing included Bayesian t-tests that enabled estimation of an effect compared to the null model^67^. For the following t-tests, Median (Mdn), confidence intervals at 95% (95% CI), the Probability of direction (Pd, representing certainty, with which an effect is in a particular direction), and BF are presented.

The Bayes Factors (BFs) are reported for the ANOVA. When BF > 1, there is evidence in favour of the alternative hypothesis compared to the null hypothesis. The higher the BF, the stronger the evidence. To appraise the obtained BF values, we followed the guidelines by Raftery^68^. For 0 < BF < 1, there is no evidence of measure difference; for 1 ≤ BF ≤ 3, evidence is anecdotal; for 3 < BF ≤ 20, evidence is substantial, and for BF > 20, evidence of difference is strong.

Further, *Bayesian correlation analysis* was used to explore the relationship between the length of each vector and participants’ age, for each RW and test order. To compare null and alternative hypotheses, we used the BF, Pd and the Highest Density Intervals at 95% (95% HDI, the shortest interval on a posterior density for a given confidence level, here 95%). This evidence is corroborated by a close-to-100% Pd and 95% HDIs that do not contain 0.

In addition, to investigate differences in Trivector measures for the three age groups at varying RWs, a *Bayesian mixed-model analysis* was performed using the brms package^69,70^. We probed five Bayesian models; weakly informative priors were used on all predictor regression coefficients (standard normal distribution for main effects and interactions). Specifically, the five (non-)linear models were computed using ‘Stan’ programming language in the brms package, which estimates parameters by applying the Hamiltonian Monte Carlo method. Four Markov chains were run, each with a warmup period of 1000 iterations and 2000 iterations used for sampling. Details that justified the model choice for the present dataset are presented in Supplementary Table S3 of Supplementary Materials. All models were compared, and the model with the best fit (highest Bayes Factor^71,72^) was retained. The chosen model was checked for convergence using Gelman Rubin statistic with convergence indicated by values close to 1.

The model formula in brms syntax is:

$$\mathrm{Thresholds}\left(\mathrm{Protan}, \mathrm{Deutan}, \mathrm{Tritan}\right)= 1+\mathrm{Age \; Group}\times \mathrm{RW}+(1|\mathrm{ID}/\mathrm{Test \; Order}).$$

The model included lengths of Protan, Deutan and Tritan vectors as dependent variables. The fixed effects of the model were comprised of two main effects (Age Group and RW), and their interaction, with three levels for each of the main effects. The random effect structure was described as follows: ID (each observer’s identifier) and Test Order (test and retest). The random effect intercepts for ID were estimated while nesting the Test Order within IDs. Main effects, their interaction and random effects were studied for each of the three dependent variables, Protan, Deutan and Tritan measures.

For the model estimates, coefficients were following the alternative hypothesis if 95% HDI, the associated 95% most likely distribution values, were non-overlapping with zero and Pd values above 98%. For each regression, the coefficient (i.e. the median estimate), the 95% HDI and the Pd are reported^71,72^. To explore the size of measure differences between the age groups, group comparisons were evaluated by generating the distribution of the modelled averages per predictor value combination; thereby, we extracted the values from the posterior fit. To evaluate the magnitude of pairwise difference between two compared subsets of measures, we considered mean difference values greater than zero. Mean, SD and confidence interval (CI) 2.5–97.5% are also reported.

Finally, to address the auxiliary aim of the study—repeatability of Trivector measures at the varying RWs, we conducted the *Bland–Altman analysis*^59^. Accordingly, estimated were test–retest mean for the vector in question ($\mathop {\text{X}}\limits^{{\prime }}$_V_) and standard deviation of the mean (SD_V_). Several further estimates were calculated to characterise the test repeatability: mean difference ($\mathop {\text{X}}\limits^{{\prime }}$_D_) and standard deviation (SD_D_) of pairwise differences across participants; upper and lower Limits of Agreement (LoAs); 95% CIs of $\mathop {\text{X}}\limits^{{\prime }}$_D_ and of upper and lower LoAs (for the formulae applied see Ref.^44^).

The main parameter, coefficient of repeatability (COR), was defined as the modulus of upper and lower LoAs, COR = 1.96 × SD_D_, adopted by the British Standards Institution^61^. According to its guidance, for a test with good repeatability, $\mathop {\text{X}}\limits^{{\prime }}$_D_ is supposed to be zero or close to zero, and 95% of the test–retest differences lie within the 95% CIs of the upper and lower LoAs^73^. Also, a test is considered to have good repeatability if no systematic learning effect is observed.

All Bayesian analyses were conducted in R (R Foundation for Statistical Computing, Vienna, Austria) with several packages used to run these: ANOVA, correlation analysis and mixed-model analysis (respectively BayesFactor^74^ and brms^69,70^). All plots were generated using the ggplot2^75^ package.

## Supplementary Information

Supplementary Information.

## Data Availability

Data analysed in this study are available at https://figshare.com/s/0f08bc3092389e5d79ab.
