# Supplementary material for: Chromatic discrimination measures in mature observers depend on the response window
Source: Sci Rep. 2022 May 31;12:9072. doi: 10.1038/s41598-022-13129-w (PMC9156755; doi:10.1038/s41598-022-13129-w)
Supplement: Supplementary file 1 — Supplementary Information. [file 41598_2022_13129_MOESM1_ESM.pdf]

Measurements of chromatic discrimination in mature observers depend on the response window

Julien Fars[1*], Thiago P. Fernandes[2], Cord Huchzermeyer[1], Jan Kremers[1], Galina V. Paramei[3*]

[1]Department of Ophthalmology, University Hospital Erlangen, Schwabachanlage 6, 91054 Erlangen, Germany

[2]Department of Psychology, Federal University of Paraiba, Cidade Universitaria S/N, 58051-900, Joao Pessoa, Brazil

[3]Department of Psychology, Liverpool Hope University, Hope Park, L16 9JD Liverpool, UK

Corresponding authors (*):

Julien Fars

Department of Ophthalmology

University Hospital Erlangen

Schwabachanlage 6

91054 Erlangen

Germany

Email: julien.fars@fau.de

Prof. Galina Paramei

Department of Psychology

Liverpool Hope University

Hope Park

L16 6JD Liverpool

United Kingdom

Email: parameg@hope.ac.uk

Supplementary Materials

Violin plots of individual Trivector measures for test and retest sessions

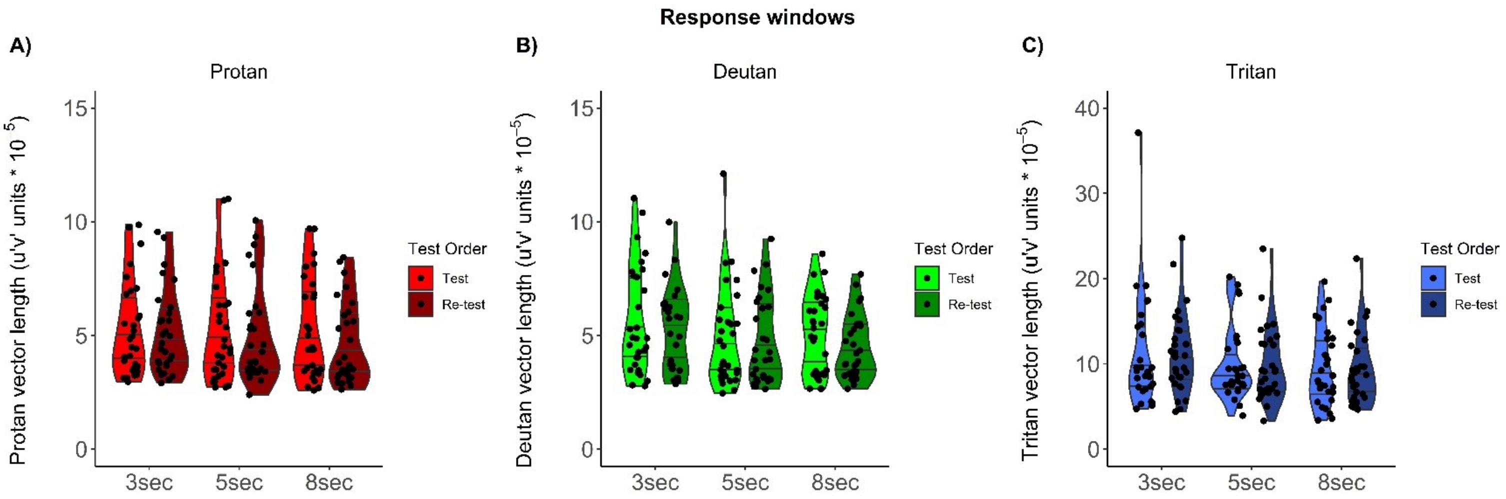

Figure S1. Violin plots representing discrimination thresholds of individual observers at the 3-sec, 5-sec and 8-sec response windows for each of the three Trivector measures: (A) Protan, (B) Deutan and (C) Tritan. Test session data are in light and Retest session data are in dark colours. Note that the y-axis scale of Tritan thresholds (C) differs from those of Protan (A) and Deutan (B) thresholds. Within violins, horizontal lines indicate 25%, 50% and 75% quantiles. The shape of the violin plots is determined using a Gaussian kernel density.

Correlation plots between the Trivector length and the participant's age

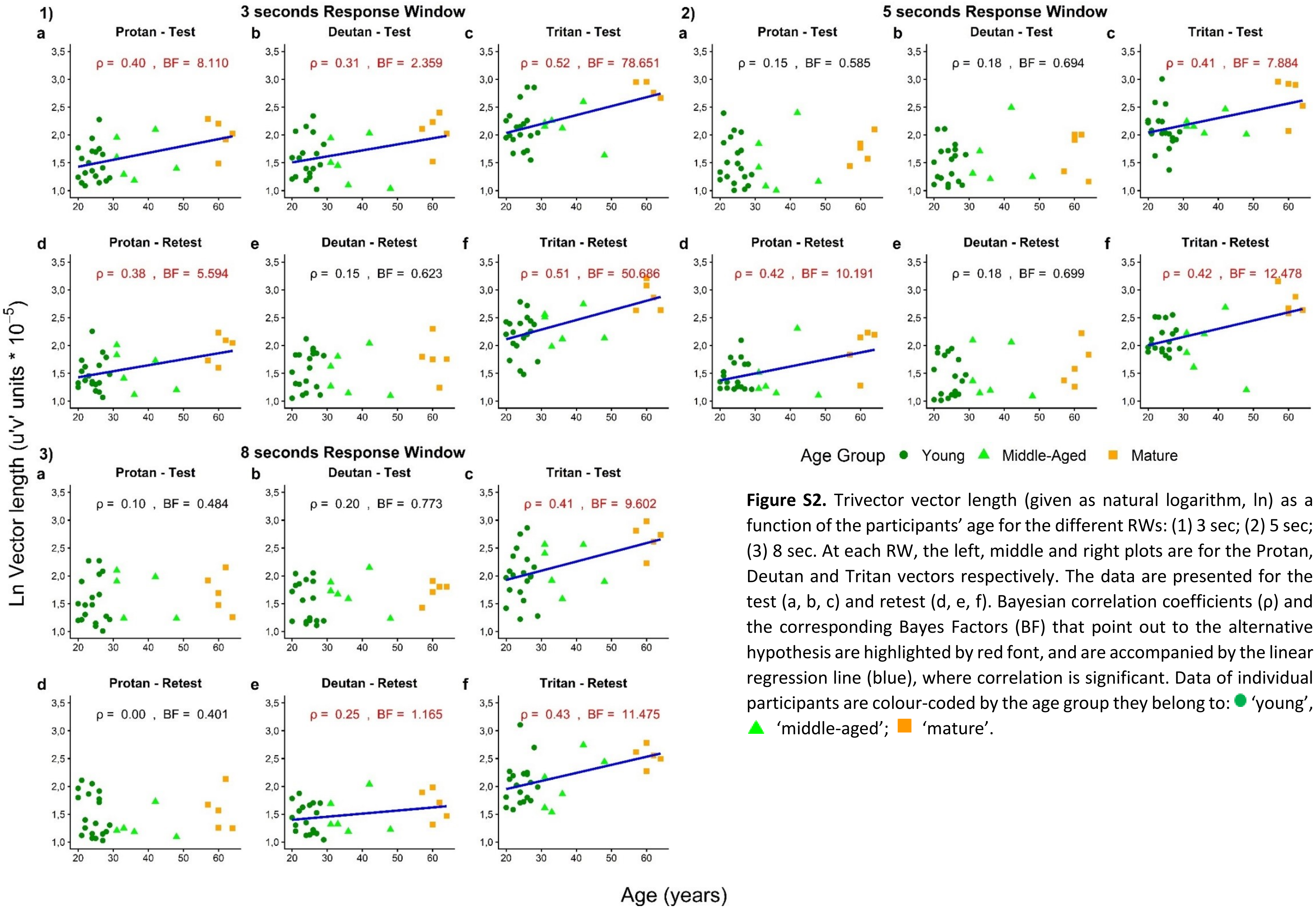

Figure S2. Trivector vector length (given as natural logarithm, ln) as a function of the participants' age for the different RWs: (1) 3 sec; (2) 5 sec; (3) 8 sec. At each RW, the left, middle and right plots are for the Protan, Deutan and Tritan vectors respectively. The data are presented for the test (a, b, c) and retest (d, e, f). Bayesian correlation coefficients (ρ) and the corresponding Bayes Factors (BF) that point out to the alternative hypothesis are highlighted by red font, and are accompanied by the linear regression line (blue), where correlation is significant. Data of individual participants are colour-coded by the age group they belong to: ● 'young', ▲ 'middle-aged'; ■ 'mature'.

Bayesian three-way ANOVA outcomes and post hoc analysis

	**Bayes Factor analysis**	
	**Bayes Factor**	**Error rate**
**RW**	0.548	±0.91%
**Vector**	**4.711×10^{83}**	±2.14%
**Test Order**	0.140	±1.1%
**RW * Vector**	0.013	±1.16%
**Vector * Test Order**	0.057	±0.57%
**RW * Test Order**	0.048	±0.75%
**RW * Vector * Test Order**	0.025	±0.44%

Table S1. Outcomes of the repeated-measures Bayesian ANOVA of the Trivector estimates, with three factors: Response Window (RW), Vector and Test Order. Effect following the alternative hypothesis is in bold.

	Bayesian Pairwise t-test – Vector						
Comparison	Median	95%CI	Pd	ROPE	% in ROPE	Prior	BF
Protan–Deutan	-1.20×10^{-3}	[-0.05; 0.05]	51.68%	[-0.04; 0.04]	86.56%	Cauchy (0 $\pm$ 0.71)	0.083
Protan–Tritan	-0.677	[0.61; 0.74]	100%	[-0.04; 0.04]	0%	Cauchy (0 $\pm$ 0.71)	**> 1000**
Deutan–Tritan	-0.678	[0.61; 0.74]	100%	[-0.04; 0.04]	0%	Cauchy (0 $\pm$ 0.71)	**> 1000**

Table S2. Outcomes of the Bayesian pairwise t-test of Vector. For each comparison calculated were median; confidence interval at 95% (95%CI); probability of direction (Pd); Region of Practical Equivalence (ROPE, a range of practically no effect); percentage of full posterior distribution inside the ROPE (% in ROPE); the type of prior distribution (Prior); and the Bayes Factor (BF). Effects that tend to follow the alternative hypothesis are highlighted in bold.

Only the Vector effect was found to follow the alternative hypothesis. Bayesian *t*-tests showed that no difference was found between Protan and Deutan vector lengths; there are, however, strong differences between each of these with Tritan vector.

Bayesian Analysis

Comparing the models

To find the model that best explains the data, we chose between five Bayesian models; weakly informative priors were used on all predictor regression coefficients (normal distribution with mean 0 and SD 1). Models were computed using the Stan programming language via the brms package which estimates parameters using Hamiltonian Monte Carlo. Four Markov chains were run, each with a warmup period of 1000 iterations and 2000 iterations used for sampling.

Model comparisons were conducted using the Bayes Factor from the package bayestestR[1,2]. The model with the highest Bayes factor is the one with the best fit.

Model M0: $Length(Protan, Deutan, Tritan) = 1 + (1|\mathrm{ID/Test\ Order})$

Model M1: $Length(Protan, Deutan, Tritan) = 1 + \mathrm{RW} + (1|\mathrm{ID/Test\ Order})$

Model M2: $Length(Protan, Deutan, Tritan) = 1 + \mathrm{Age\ Group} + (1|\mathrm{ID/Test\ Order})$

Model M3: $Length(Protan, Deutan, Tritan) = 1 + \mathrm{Age\ Group} + \mathrm{RW} + (1|\mathrm{ID/Test\ Order})$

Model M4: $Length(Protan, Deutan, Tritan) = 1 + \mathrm{Age\ Group} \times \mathrm{RW} + (1|\mathrm{ID/Test\ Order})$

Bayes Factors for Model Comparison

	Numerator				
Denominator	M1	M2	M3	M4	M0
M1	1	1.47e+09	1.42e+11	9.53e+19	0.249
M2	6.81e-10	1	96.57	6.48e+10	1.69e-10
M3	7.05e-12	0.010	1	6.71e+08	1.75e-12
M4	1.05e-20	1.54e-11	1.49e-09	1	2.61e-21
M0	4.02	5.91e+09	5.71e+11	3.83e+20	1

Table S3. Model comparison. All models from the simplex to the more complex one were compared. A higher value (of Bayes Factor) indicated a better fit of the numerator model over the denominator model. The comparisons show the best fit comes from model M4.

The numerators are tested against denominators. Among our 5 tested models, it is apparent that the best fitting model is M4 model.

Convergence was checked using Gelman Rubin statistic with convergence indicated by values close to 1. All values from the parameters were equal to 1 or 1.01 (Fig. S3). The models were compared, and model M4 was considered suitable for our study; its convergence was checked.

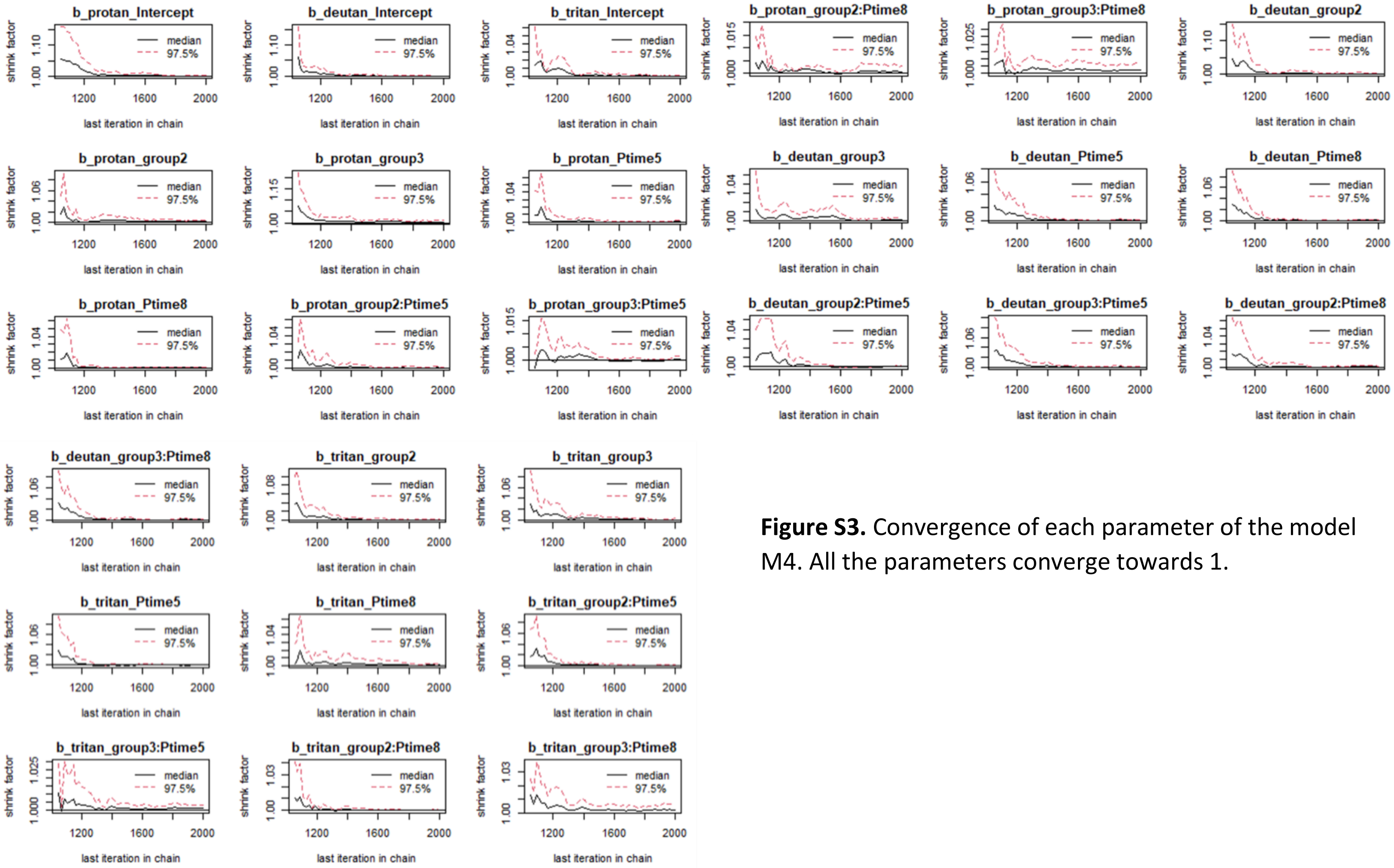

Figure S3. Convergence of each parameter of the model M4. All the parameters converge towards 1.

The model summary

When 0 is not included between the Highest Density Intervals (95%HDI) then we can assume decent effect of the parameter. These parameters are indicated in bold in the following table. The 95%HDI and its corresponding probability of direction for each parameter are indicated on Table S4.

Parameter	Vector	Estimate	95%HDI	Pd (Probablity of direction)
**Intercept**	**Protan**	**4.59**	**[3.86, 5.34]**	**100%**
Middle-aged	Protan	0.48	[-1.08, 1.97]	73.50%
**Mature**	**Protan**	**2.75**	**[1.03, 4.30]**	**99.90%**
RW 5sec	Protan	0.03	[-0.66, 0.72]	52.48%
RW 8sec	Protan	0.23	[-0.47, 0.93]	74.62%
Middle-aged * RW 5sec	Protan	-0.27	[-1.63, 1.18]	64.00%
Mature * RW 5sec	Protan	-0.77	[-2.33, 0.73]	83.97%
Middle-aged * RW 8sec	Protan	-0.91	[-2.28, 0.60]	89.30%
**Mature * RW 8sec**	**Protan**	**-2.17**	**[-3.59, -0.56]**	**99.83%**
**Intercept**	**Deutan**	**5.11**	**[4.42, 5.82]**	**100%**
Middle-aged	Deutan	-0.31	[-1.71, 1.20]	67.15%
**Mature**	**Deutan**	**2.07**	**[0.57, 3.56]**	**99.30%**
RW 5sec	Deutan	-0.64	[-1.31, 0.02]	97.00%
**RW 8sec**	**Deutan**	**-0.70**	**[-1.38, -0.03]**	**97.62%**
Middle-aged * RW 5sec	Deutan	0.84	[-0.47, 2.22]	88.83%
Mature * RW 5sec	Deutan	-0.89	[-2.30, 0.61]	88.40%
Middle-aged * RW 8sec	Deutan	1.04	[-0.36, 2.38]	92.70%
Mature * RW 8sec	Deutan	-0.88	[-2.31, 0.57]	88.55%
**Intercept**	**Tritan**	**9.17**	**[7.77, 10.57]**	**100%**
Middle-aged	Tritan	0.74	[-2.31, 3.69]	69.00%
**Mature**	**Tritan**	**10.56**	**[7.27, 13.62]**	**100%**
RW 5sec	Tritan	-0.58	[-1.89, 0.80]	79.97%
RW 8sec	Tritan	-0.93	[-2.20, 0.46]	91.90%
Middle-aged * RW 5sec	Tritan	-0.89	[-3.58, 1.80]	73.78%
**Mature * RW 5sec**	**Tritan**	**-3.22**	**[-6.42, -0.42]**	**98.08%**
Middle-aged * RW 8sec	Tritan	-0.03	[-2.80, 2.64]	51.30%
**Mature * RW 8sec**	**Tritan**	**-4.90**	**[-7.85, -2.02]**	**99.90%**

Table S4. Raw output of the model M4. All parameters are indicated with their corresponding highest density values (95%HDI) and probability of direction (Pd) for each vector. Intercepts and parameters that tend to follow the alternative hypothesis are indicated in bold.

When evaluating the model, it is important to remember that we are using categorical variables and not continuous variables. It means that we do not have a grand mean we can compare the variables to. The intercepts for each vector (Protan, Deutan & Tritan) corresponds to the value of the vector at 3sec for the 'young' age group. Moreover, the estimated values are compared to their local intercepts. For example, when looking at the value of the Mature group at 8sec RW for Tritan (bottom line in Table S4), the estimate is in fact compared to its local intercept value i.e. the 'mature' group at 3-sec RW.

Every “missing” line is in fact the local intercept (e.g. the ‘young’ group at the 3-sec RW condition). In order to fully appreciate the results from the model, another table has been added (Table S5), showing the results when the mature group and the 8sec RW condition were used as the baseline. The model used to get the Table S5 is the same as before (model M4) although we recoded the data, so the RW condition and the age group use different baseline factors (i.e., 3-sec RW @ ‘young’ group). For more clarity, we refer to this model as “model M4 recoded”.

Parameter	Vector	Estimate	95% HDI	Pd (Probability of direction)
**Intercept**	**Protan**	**5.44**	**[4.07, 6.83]**	**100%**
Young	Protan	-0.61	[-2.20, 0.95]	77.12%
Middle-aged	Protan	-1.04	[-2.89, 0.92]	85.25%
**RW 3 sec**	**Protan**	**1.93**	**[0.63, 3.28]**	**99.78%**
RW 5 sec	Protan	1.19	[-0.13, 2.56]	96.30%
**Young * RW 3 sec**	**Protan**	**-2.15**	**[-3.66, -0.67]**	**99.85%**
Middle aged * RW 3 sec	Protan	-1.25	[-3.14, 0.49]	91.22%
Young * RW 5 sec	Protan	-1.39	[-2.87, 0.11]	96.75%
Middle-aged * RW 5 sec	Protan	-0.77	[-2.54, 1.08]	80.12%
**Intercept**	**Deutan**	**5.59**	**[4.32, 7.02]**	**100%**
Young	Deutan	-1.17	[-2.80, 0.24]	94.03%
Middle aged	Deutan	-0.45	[-2.37, 1.33]	67.73%
**RW 3 sec**	**Deutan**	**1.57**	**[0.21, 2.88]**	**99.20%**
RW 5 sec	Deutan	0.04	[-1.27, 1.26]	52.50%
Young * RW 3 sec	Deutan	-0.87	[-2.31, 0.62]	87.35%
Middle-aged * RW 3 sec	Deutan	-1.90	[-3.71, -0.14]	98.05%
Young * RW 5 sec	Deutan	0.02	[-1.44, 1.41]	51.23%
Middle-aged * RW 5 sec	Deutan	-0.18	[-1.88, 1.64]	58.03%
**Intercept**	**Tritan**	**13.86**	**[11.09, 16.80]**	**100%**
**Young**	**Tritan**	**-5.67**	**[-8.82, -2.40]**	**99.92%**
**Middle-aged**	**Tritan**	**-4.97**	**[-8.80, -0.93]**	**99.30%**
**RW 3 sec**	**Tritan**	**5.87**	**[3.22, 8.65]**	**100%**
RW 5 sec	Tritan	2.04	[-0.51, 4.86]	93.27%
**Young * RW 3 sec**	**Tritan**	**-4.91**	**[-8.09, -1.98]**	**99.98%**
**Middle-aged * RW 3 sec**	**Tritan**	**-4.87**	**[-8.34, -1.20]**	**99.70%**
Young * RW 5 sec	Tritan	-1.66	[-4.77, 1.21]	85.85%
Middle-aged * RW 5 sec	Tritan	-2.56	[-6.03, 1.02]	91.45%

Table S5. Raw output of the recoded model M4 recoded. All parameters are indicated with their corresponding highest density values (95%HDI) and probability of direction (Pd) for each vector. Intercepts and parameters that follow the alternative hypothesis are indicated in bold.

As we can see in the Table S5, the results from the model M4 recoded completes well the results displayed in Table S4.

Exploring the power: Simulation

We admit that our subsamples 'middle-aged' and 'mature' participants are small. Since our study is a pilot, we estimated the power of our Bayesian mixed-model analysis. We present here simulations of analysis outcomes while using different effect sizes for the parameters we are exploring. Although the term "power analysis" is not used in relation to the Bayesian framework, we employ it here as a proxy notion of a measure that allows us to assess how reliably our outcomes can be explained by the model with varying parameters. Importantly, the two parameters that can affect the analysis "power" are the sample size and the effect size. Different stages of analysis presented below follows the guidance of Kruschke (2011)[3]and indications about the *brms* package[4,] made by Kurz[5].

To explore the "power" within the Bayesian framework, our first step was to (i) use prior information to specify hypothetical distribution of all parameter values in the model, and (ii) use the so estimated distribution to generate synthetic data that would be similar to our actual sample's data; to then (iii) fit the model using the synthetic data and (iv) examine the posterior to consider if we achieved our goal. To estimate the "power", we had to (v) reiterate the latter procedure many times.

Here we illustrate only the "power" analysis for the Protan estimates of the 'mature' group at 8-sec RW. Among the effects we illustrate in our study, the Protan estimate for 'mature' participants at 8-sec RW is lower than their Tritan estimates at either 5-sec RW or at 8-sec RW (see Table S4). This makes a good example.

First, we simulated a dataset that follows our actual subsamples (N = 19 for 'young', N = 6 for 'middle-aged', and N = 5 for 'mature'). To study the impact of the effect size, we tested the options representing the three effect size ranges: a low effect size (Hedges' g = 0.2), a medium effect size (Hedges' g = 0.5), and a large effect size (Hedges' g = 1). We found that the latter approximated our actual data in the best way.

The model described in the Methods section was fitted by us to 100 datasets for each of the so determined effect sizes by using pseudorandom generated values that follow the subsample size and the parameters of the study.

From all these model simulations, we extorted the searched estimates, and compared these to the idealised effect sizes (either 0.2, 0.5 or 1) keeping in mind the null hypothesis. The model "power" was estimated by determining of how many fits an estimate had, whose 95% of the values of the distribution were not overlapping with 0 (i.e. following the 95%HDI method we present in our Methods section).

For the given distribution of the participants, we found that the "power" estimation was quite high despite low number of the participants (see Fig. S4).

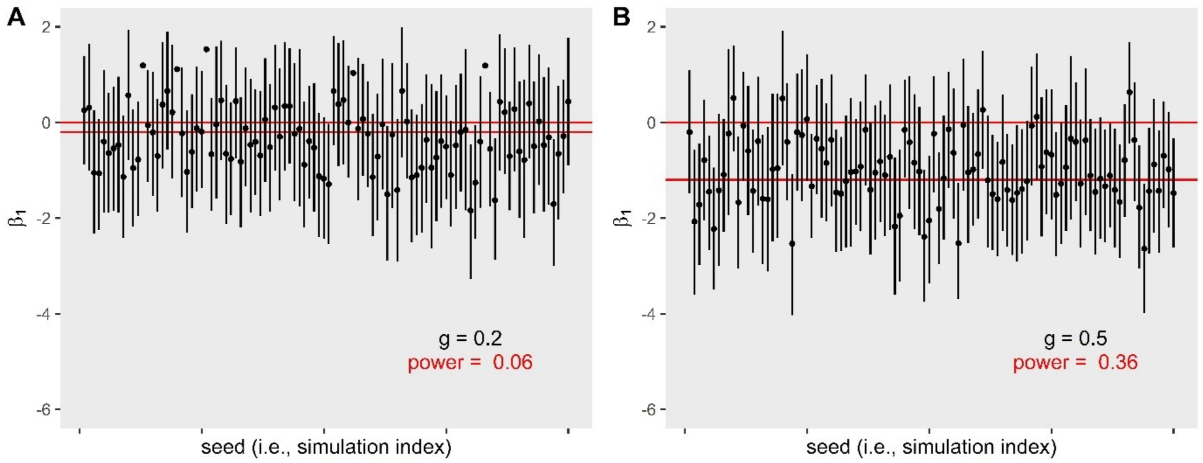

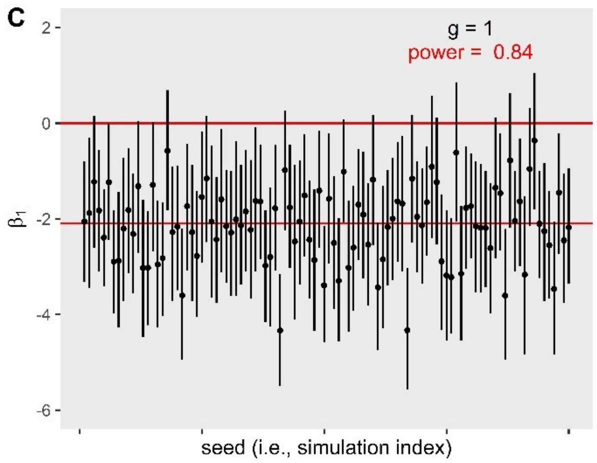

Figure S4. Power estimations of the Protan estimates of the model for three defined effect sizes (A) *g* = 0.2, (B) *g* = 0.5, (C) *g* = 1. The horizontal lines show the idealized estimates (-0.2, -1.2 and -2.1 respectively) and the null hypothesis (0)

However, this procedure is close to a Frequentist framework procedure and may not reflect the "power" that could be attributed to Bayesian analysis. Further analysis of precision may be necessary in other studies with small samples.

Bland-Altman analysis of repeatability of Trivector measures at the varying response windows

For all three tested response windows (RWs) and Trivector measures, mean test-retest differences ($\acute{X}_D$) are close to zero (Table S6 and Fig. S5). This corroborates outcomes of the ANOVA in relation to the Test Order in that there are no systematic differences between test and retest estimates, i.e. no learning effect. As is apparent from Table S6 and Fig. S5, for all RW conditions and each vector, the upper and lower limits of agreement (LoAs) are comparable. The coefficients of repeatability (CORs) for all three vectors and all RW conditions are slightly greater than the corresponding estimates reported by Fernandes et al.[6], for 6-sec and 8-sec RWs, which probably results from a relatively small participant sample in the present study (N=30) compared to significantly greater participant numbers in the two samples in the Fernandes et al. (N=111 and N=79). Note that Table 2 in Ref. [6] shows Trivector measures in 10^{-4} u'v' units compared to 10^{-5} u'v' units adopted in the CCT generation employed in the present study). Here we also observe that for Protan and Deutan measures, COR values vary slightly but not systematically between the three RW conditions; in comparison, for the Tritan measure, the COR value decreases with the RW extension indicating a progressively lower test–retest variability of participants' responses a longer exposure to the stimulus.

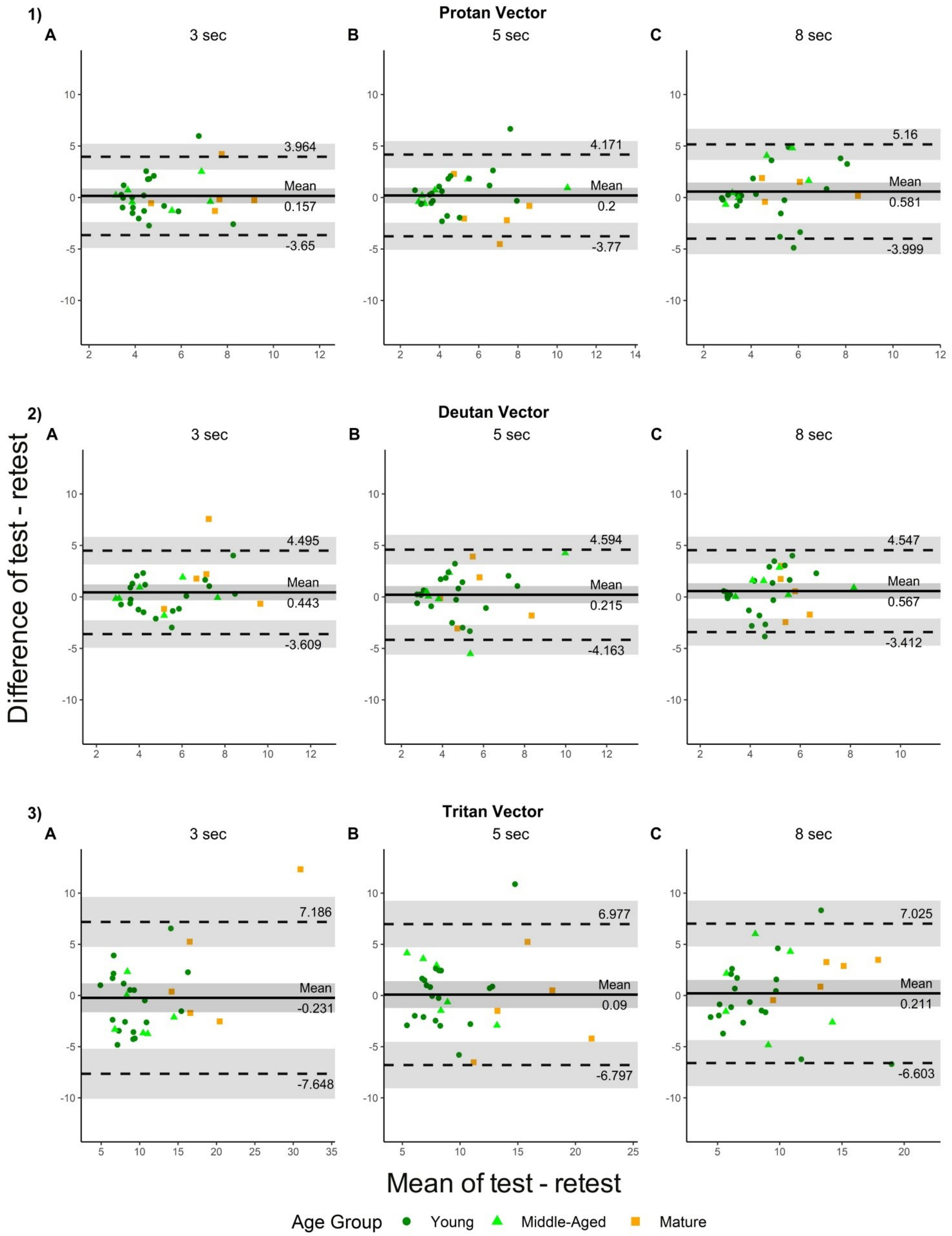

Figure S5. Bland-Altman plots for the varying response windows: (A) 3 sec, (B) 5 sec, and (C) 8 sec for (1) Protan, (2) Deutan, and (3) Tritan vectors. Data of individual participants are colour-coded according to the age group they belong to. Mean of test-retest differences and limits of agreements (LoA) are indicated by solid and dashed lines respectively; SD of mean and CI of upper and lower LoAs are indicated by the shadowed regions. Note that data are presented in 10^{-5} u'v' units adopted in the CCT generation employed in the present study here compared to Trivector measures data 10^{-4} u'v' units in Table 2 in Ref. 3.

Test-Retest Statistic	Response window		
	**3 sec**	**5 sec**	**8 sec**
**Protan**			
$\acute{X}_V$ (SD_V)	5.161 (1.691)	5.010 (1.963)	4.854 (1.630)
$\acute{X}_D$ (SD_D)	0.157 (1.942)	0.200 (2.026)	0.580 (2.336)
95% CI $\acute{X}_D$	0.880–(-0.567)	0.955–(-0.554)	1.451–(-0.290)
Upper LoA	3.964	4.171	5.160
95% CI Upper LoA	5.217–2.711	5.477–2.864	6.667–3.653
Lower LoA	-3.650	-3.770	-3.999
95% CI Lower LoA	(-2.397)–(-4.903)	(-2.463)–(-5.076)	(-2.492)–(-5.506)
**COR**	**3.806**	**3.970**	**4.578**
**Deutan**			
$\acute{X}_V$ (SD_V)	5.398 (1.835)	4.780 (1.718)	4.760 (1.180)
$\acute{X}_D$ (SD_D)	0.443 (2.067)	0.215 (2.234)	0.567 (2.030)
95% CI $\acute{X}_D$	1.213–(-0.328)	1.047–(-0.616)	1.323–(-0.189)
Upper LoA	4.495	4.594	4.546
95% CI Upper LoA	5.828–3.161	6.035–3.153	5.856–3.237
Lower LoA	-3.609	-4.163	-3.412
95% CI Lower LoA	(-2.276)–(-4.943)	(-2.822)–(-5.604)	(-2.102)–(-4.722)
**COR**	**4.051**	**4.379**	**3.979**
**Tritan**			
$\acute{X}_V$ (SD_V)	11.095 (5.380)	9.800 (3.875)	9.333 (3.908)
$\acute{X}_D$ (SD_D)	-0.231 (3.780)	0.090 (3.514)	0.211 (3.477)
95% CI $\acute{X}_D$	1.178–(-1.640)	1.399–(-1.218)	1.506–(-1.084)
Upper LoA	7.186	6.977	7.025
95% CI Upper LoA	9.628–4.745	9.244–4.711	9.268–4.783
Lower LoA	-7.648	-6.797	-6.603
95% CI Lower LoA	(-5.207)–(-10.089)	(-4.530)–(-9.064)	(-4.360)–(-8.846)
**COR**	**7.409**	**6.887**	**6.815**

Table S6. Outcomes of the Bland-Altman analysis. Test-retest parameters of the Trivector measures (10^{-5} u'v' units) for 3-sec, 5-sec and 8-sec response windows: X'_V, mean for the vectors; SD_V, standard deviations of the vectors; X'_D, mean for the difference between pairs of the vectors; SD_D, standard deviations of the mean difference; LoA, limits of agreement; CI, confidence intervals; COR, coefficient of repeatability[7].

References

1. Makowski, D., Ben-Shachar, M. S., Chen, S., & Lüdecke, D. Indices of effect existence and significance in the Bayesian framework. *Front. Psychol.* **10**: 2767. https://doi.org/10.3389/fpsyg.2019.02767 (2019).
2. Makowski, D., Ben-Shachar, M. S., & Lüdecke, D. bayestestR: describing effects and their uncertainty, existence and significance within the Bayesian framework. *J. Open Source Soft.* **4**(40): 1541. https://doi.org/10.21105/joss.01541 (2019).
3. Kruschke, J. K. *Doing Bayesian Data Analysis: A tutorial with R and BUGS* (Academic Press, Burlington, MA, 2011).
4. Bürkner, P. C. brms: An R package for bayesian multilevel models using Stan. *J. Stat. Softw.* **80**(1): 1-28. https://doi.org/10.18637/jss.v080.i01 (2017).
5. Kurz, S. *Doing Bayesian Data Analysis in brms and the tidyverse*. Available from: https://bookdown.org/ajkurz/DBDA_recoded/ (2019).
6. Fernandes, T. M. P., Santos, N. A., & Paramei, G. V. Cambridge Colour Test: Reproducibility in normal trichromats. *J. Opt. Soc. Am. A* **37**(4): A70-A80. https://doi.org/10.1364/JOSAA.380306 (2020).
7. Bland, M. J., & Altman, D. Statistical methods for assessing agreement between two methods of clinical measurement. *Lancet* **327**(8476): 307-310. https://doi.org/10.1016/S0140-6736(86)90837-8 (1986).
